# Prophylactic Proton Pump Inhibitors in Critically Ill Patients Undergoing Invasive Ventilation: A Systematic Review and Meta‐Analysis of Randomized Trials

**DOI:** 10.1155/ccrp/5581019

**Published:** 2026-03-24

**Authors:** Jinlu Hu, Haiyan Ye, Bo Li, Xuemei Zheng

**Affiliations:** ^1^ Department of Adult Intensive Care Unit, Chengdu Women’s and Children’s Central Hospital, School of Medicine, University of Electronic Science and Technology of China, Chengdu, 611731, China, uestc.edu.cn

**Keywords:** *Clostridioides difficile* infection, critically ill, invasive ventilation, proton pump inhibitors, upper gastrointestinal bleeding, ventilator-associated pneumonia

## Abstract

**Objective:**

To assess the safety and efficacy of prophylactic proton pump inhibitors (PPIs) among critically ill adults undergoing invasive ventilation.

**Methods:**

We systematically searched PubMed, Web of Science, Embase, and the Cochrane Library for randomized controlled trials (RCTs) published up to September 19, 2024. Eligible trials compared prophylactic PPIs with placebo or no prophylaxis for upper gastrointestinal bleeding (UGIB) prevention in adults receiving invasive ventilation. Nine RCTs encompassing 8388 patients were included. Data were pooled using Review Manager 5.4, with a random‐effects model applied to all outcomes.

**Results:**

All‐cause mortality rates were comparable between the PPI and control groups (odds ratio [OR]: 0.98; 95% confidence interval [CI]: 0.89−1.07; I^2^ = 0%; *p* = 0.61). PPI prophylaxis significantly reduced the incidence of UGIB (OR: 0.42; 95% CI: 0.23−0.75; I^2^ = 33%; *p* = 0.003) and clinically important UGIB (OR: 0.36; 95% CI: 0.19−0.69; I^2^ = 10%; *p* = 0.002). The analysis revealed no significant increase in the risk of ventilator‐associated pneumonia (OR, 0.99; 95% CI, 0.87 to 1.12; I^2^ = 0%; *p* = 0.86) or *Clostridioides difficile* infection (OR, 1.63; 95% CI, 0.97 to 2.73; I^2^ = 0%; *p* = 0.07) with PPI use. Durations of intensive care unit stay and invasive ventilation did not differ significantly between groups.

**Conclusion:**

Among mechanically ventilated patients, prophylactic PPI therapy significantly decreased UGIB and clinically important UGIB risk compared to placebo, without affecting all‐cause mortality.

**Highlights**
• PPIs significantly reduced the risk of UGIB and clinically important UGIB in critically ill adults undergoing invasive ventilation.
• No significant increase in mortality, *Clostridioides difficile* infection, or ventilator‐associated pneumonia was found with PPI prophylaxis.
• The findings support the rational use of PPIs for stress ulcer prophylaxis in critically ill, mechanically ventilated adults.
• Future studies should refine patient selection and optimize dosing regimens.

## 1. Introduction

Critically ill patients face a substantial risk of upper gastrointestinal bleeding (UGIB) secondary to stress‐related mucosal damage from gastrointestinal hypoperfusion and ischemia‐reperfusion injury [1]. While initially superficial and clinically silent, these lesions may progress to involve the submucosa and muscularis propria, potentially eroding major vessels and leading to clinically important UGIB [2]. The occurrence of clinically important UGIB is low, at around 1.1%–2.6% [3–5], but when it occurs, it can be serious. A multicenter study by Cook et al. (*n* = 1666) revealed that clinically important UGIB contributed to morbidity and mortality, with a relative risk (RR) of death ranging from 1.0 to 4.1 across different analytical approaches and an additional 4–8 days in intensive care unit (ICU) stay [6].

To prevent UGIB, clinicians often prescribe prophylactic acid suppressants, most commonly proton pump inhibitors (PPIs) [7–9]. PPIs suppress gastric acid secretion and raise gastric pH by irreversibly blocking the H^+^/K^+^‐ATPase in gastric parietal cells [10]. Histamine‐2 receptor antagonists (H2RAs) provide an alternative approach; however, PPIs achieve more potent and sustained elevation of gastric pH than H_2_RAs [11, 12], thereby conferring superior protection against gastric mucosal injury. Current clinical guidelines also recommend that critically ill patients at high risk for gastrointestinal bleeding should receive PPI prophylaxis [13–15].

However, the role of prophylactic PPIs in critically ill adults remains debated. In a large, multicenter randomized controlled trial (RCT) known as SUP‐ICU, investigators demonstrated that although pantoprazole reduced clinically important UGIB risk versus placebo, it paradoxically increased mortality in the most severely ill patient subgroup [16, 17]. In contrast, the recent REVISE trial showed significant UGIB reduction in mechanically ventilated patients without effect on 90‐day mortality [18]. Moreover, PPIs have been linked to several adverse events, including pneumonia and *Clostridioides difficile* infection [19, 20], which are of particular concern in patients undergoing invasive ventilation.

Therefore, the results of clinical trials on the prophylactic use of PPIs are inconsistent, highlighting the need for high‐quality systematic reviews and meta‐analyses. These efforts aim to comprehensively assess the efficacy and safety of PPIs and provide important evidence for their rational use.

## 2. Methods

This systematic review was registered with PROSPERO (CRD42024588496). We conducted and reported this review according to the Preferred Reporting Items for Systematic Reviews and Meta‐Analyses (PRISMA) guidelines [21].

### 2.1. Data Sources and Searches

A research librarian (X. Z.) who is familiar with this topic codeveloped the search strategy. Two reviewers (J. H. and H. Y.) independently searched PubMed, Web of Science, Embase, and the Cochrane Library for studies published up to September 19, 2024. Search terms included “critical illness,” “critically ill,” “ICU,” “mechanical ventilation,” “proton pump inhibitors,” “PPI,” “pantoprazole,” “omeprazole,” “lansoprazole,” “rabeprazole,” “esomeprazole,” and “randomized controlled trial.”

### 2.2. Study Selection

To identify eligible studies, two reviewers (J. H. and H. Y.) screened the titles and abstracts independently and then reviewed the full texts. Any disagreements were resolved through discussion or, if necessary, by a third adjudicator (B. L.). We included RCTs that compared the prophylactic use of PPIs for UGIB with placebo or no prophylaxis in adults undergoing invasive ventilation. Eligible trials reported any of the following outcomes: all‐cause mortality at any time point, UGIB, clinically important UGIB, *Clostridioides difficile* infection, ventilator‐associated pneumonia (VAP), duration of ICU stay, and duration of invasive ventilation. There were no restrictions on the type, dose, or route of PPI administration.

### 2.3. Data Extraction

Two reviewers (J. H. and H. Y.) extracted data from each eligible trial, with discrepancies adjudicated by a third reviewer (B. L.). The following information was recorded in Excel spreadsheets: study characteristics (first author, publication year, and country), population characteristics (sample size, age, APACHE II score, and inclusion criteria), intervention and comparator details (agent name, dose, route, frequency, and duration), and outcomes and their definitions.

### 2.4. Outcome Measures

We abstracted the following outcomes, along with their definitions and time frames:

1. All‐cause mortality (longest follow‐up to 90 days).
2. UGIB as defined by trial authors. We accepted diverse definitions for UGIB, including hematemesis, coffee‐ground emesis, nasogastric aspirate with frank blood or coffee‐ground appearance, melena, or hematochezia.
3. Clinically important UGIB as defined by trial authors. We accepted definitions that included evidence of UGIB accompanied by any of the following: significant hemodynamic changes unexplained by other causes, significant decrease in hemoglobin concentration, transfusion of ≥ 2 units of red blood cells, and need for upper gastrointestinal endoscopy or surgery.
4. VAP based on the diagnostic criteria used in each trial.
5. *Clostridioides difficile* infection as reported by trial authors.
6. Duration of ICU stay and invasive ventilation in days.

### 2.5. Risk of Bias Assessment

Methodological quality was assessed using the Cochrane Risk of Bias tool [22]. Two reviewers (J. H. and H. Y.) independently evaluated the key domains: randomization, allocation concealment, blinding, incomplete outcome data, selective reporting, and other biases. Risk levels for each domain were categorized as low, unclear, or high.

### 2.6. Data Analysis

We performed statistical analyses using Review Manager 5.4. Pooled estimates were derived from a random‐effects model. Dichotomous outcomes were synthesized as odds ratios (ORs) with 95% confidence intervals (CIs). Continuous outcomes measured on consistent scales were reported as mean differences (MDs) with 95% CIs. Data initially expressed as median and interquartile range were recalculated as mean and standard deviation when applicable, following the guidelines in The Cochrane Handbook. Heterogeneity assessment utilized the I^2^ statistic. When substantial heterogeneity (I^2^ > 50%) was detected, we performed sensitivity analyses to explore potential sources. Statistical significance was defined as *p* < 0.05.

## 3. Results

**FIGURE 1:**
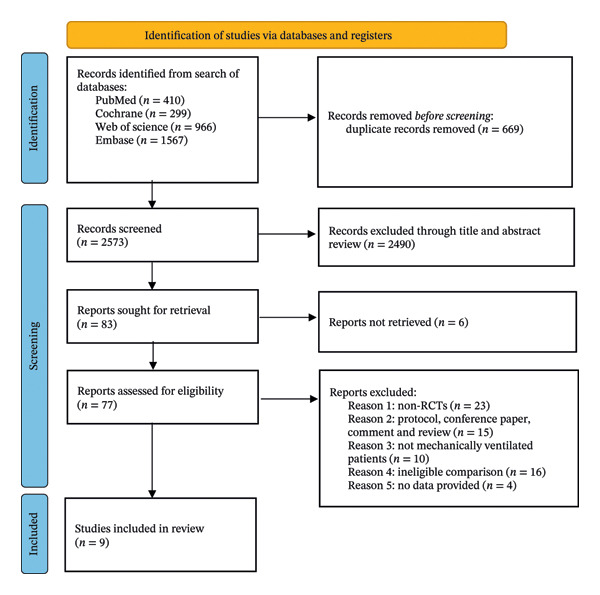
Flow diagram of studies identified in the systematic review. RCTs = randomized clinical trials.

Figure 1 summarizes the flow of study selection. Searching electronic databases retrieved 3242 items, of which 669 were in duplicate. After title and abstract screening, reviewers assessed 77 full texts. Eventually, nine RCTs were included in the quantitative analysis [16, 18, 23–29].

### 3.1. Study Characteristics

Nine RCTs were included, of which three [16, 18, 23] were multicenter trials. A total of 8388 ICU patients were enrolled, with sample sizes ranging from 91 to 4821. The geographical distribution of the study population was extensive, originating from 18 different countries, including the United States, Australia, England, Canada, China, and Brazil. Regarding disease severity, five studies reported mean APACHE II scores, all of which were above 15, reflecting a very high severity of the disease in the study population. In terms of interventions, five studies [16, 18, 23, 24, 29] utilized pantoprazole at a dosage of 40 mg intravenous (IV) daily, three studies [25, 27, 28] employed omeprazole IV at varying dosages, and the remaining study [26] used lansoprazole 30 mg administered by a nasogastric tube. The characteristics of the included studies are shown in Table 1.

**TABLE 1:** Baseline characteristics of the included trials.

Author	Single vs multicenter	Country	Total patients (I/C)	Average age (years) (I/C)	Enrollment period	Characteristics of patients	APACHE II score (I/C)	Intervention
Alhazzani 2017	Multicenter	Canadian, Saudi Arabian, and Australian	49/42	61.8 (48.4–73.5)/55.3 (42.4–65.6)	May 2015 to December 2015	Adults MV ≥ 48 h	21 (17–26)/21.5 (14–27)	Pantoprazole 40 mg IV daily
Cook 2024	Multicenter	Australia, Brazil, Canada, England, Kuwait, Pakistan, and Saudi Arabia	2417/2404	58.2 ± 16.4/58.3 ± 16.4	July 9, 2019, to October 30, 2023	Adults were undergoing MV in the ICU	21.8 ± 8.4/21.7 ± 8.2	Pantoprazole 40 mg IV daily
El‐Kersh 2018	Single	United States	55/47	62 (49.5–68)/58 (40.5–66.5)	July 2013 to September 2016	Adults MV > 48 h with no contraindications to enteral nutrition	NA	Pantoprazole 40 mg IV daily
Kantorova 2004	Single	Czech Republic	72/75	44 ± 15/46 ± 19	February 2000 to June 2002	Adults in the surgical ICU with risk factors for UGIB	17.5 ± 8.6/18.1 ± 9.3	Omeprazole 40 mg IV daily
Krag 2018	Multicenter	Denmark, Finland, the Netherlands, Norway, Switzerland, and the United Kingdom	1272/1310	67 (56–75)/67 (55–75)	January 4, 2016, to October 22, 2017	Adults admitted to the ICU with at least one risk factor for UGIB	NA	Pantoprazole 40 mg IV daily
Lin 2016	Single	Taiwan, China	60/60	66.7 ± 16.8/64.8 ± 18.6	June 1, 2009, to February 29, 2012	Adults MV > 48 h and were prepared to be weaned from the ventilator	21.3 ± 6.7/19.9 ± 6.9	Lansoprazole 30 mg administered by nasogastric tube
Liu 2013	Single	China	58/53		April 2006 to December 2008	Adults with intracranial hemorrhage in neurosurgical ICU	NA	Omeprazole 40 mg IV twice daily
Oliynyk 2021	Single	Ukraine	100/100	48.6 ± 7.6/46.3 ± 6.8	2018 to 2019	Patients with severe craniocerebral injury were on prolonged MV	22.3 ± 1.4/24.8 ± 1.7	Omeprazole 0.2 mg/kg IV daily
Selvanderan 2016	Single	Australia	106/108	52 (18)/52 (17)	January 28, 2014, and January 27, 2015	Adult MV > 24 h and receiving enteral nutrition	NA	Pantoprazole 40 mg IV daily

*Note:* I, intervention group; C, control group; IV, intravenous; UGIB, upper gastrointestinal bleeding.

Abbreviations: APACHE II, Acute Physiology and Chronic Health Evaluation II; ICU, intensive care unit; MV, mechanical ventilation; NA, not applicable.

### 3.2. Risk of Bias

**FIGURE 2:**
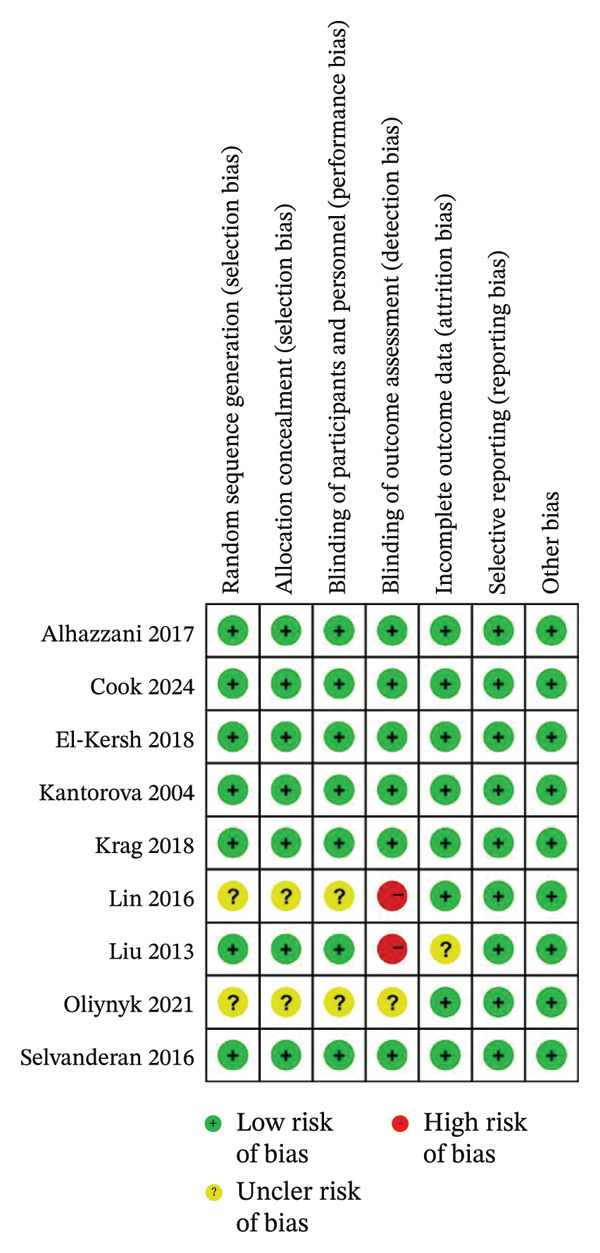
Risk of bias.

A summary of the risk of bias is presented in Figure 2. Seven studies [16, 18, 23–25, 27, 29] exhibited low risk of selection bias through adequate descriptions of randomization and concealment, whereas the remaining two studies [26, 28] were unclear. No studies were at high risk of performance bias; however, two studies [26, 28] were classified as unclear due to insufficient detail provided by the authors. Two studies [26, 27] were judged to be at high risk of detection bias. Most studies showed low risk of attrition, reporting, and other biases.

## 4. Outcomes

### 4.1. All‐Cause Mortality

**FIGURE 3:**
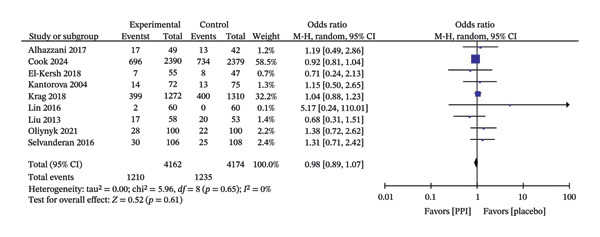
Forest plot for all‐cause mortality.

All included studies reported all‐cause mortality, and follow‐up times ranged from hospital discharge to 90 days. Subsequent analysis revealed no statistically significant difference in all‐cause mortality between the PPI and control groups (OR: 0.98; 95% CI: 0.89−1.07; I^2^ = 0%; *p* = 0.61) (Figure 3).

### 4.2. UGIB

**FIGURE 4:**
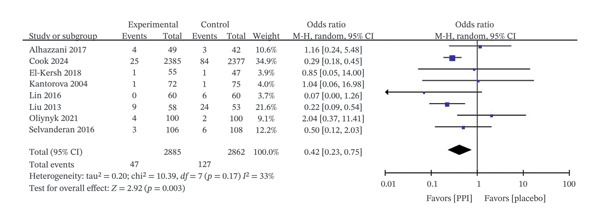
Forest plot for upper gastrointestinal bleeding.

**FIGURE 5:**
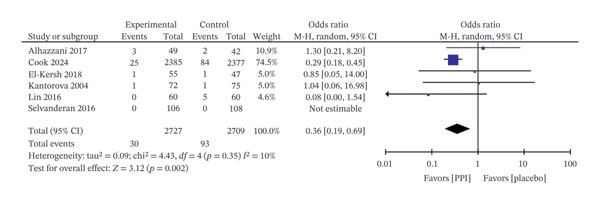
Forest plot for clinically important upper gastrointestinal bleeding.

Eight studies [18, 23–29] enrolling 5747 patients reported UGIB. The incidence of UGIB was significantly lower in the PPI group than in the control group (OR: 0.42; 95% CI: 0.23−0.75; I^2^ = 33%; *p* = 0.003) (Figure 4). Meanwhile, six studies [18, 23–26, 29] assessed clinically important UGIB, revealing that PPIs significantly reduced its occurrence (OR: 0.36; 95% CI: 0.19−0.69; I^2^ = 10%; *p* = 0.002) (Figure 5).

### 4.3. VAP and *Clostridioides Difficile* Infection

**FIGURE 6:**
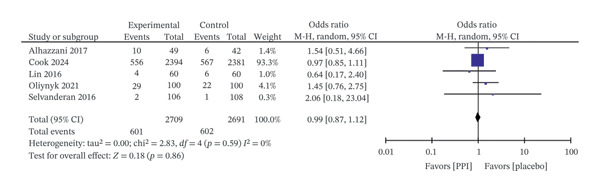
Forest plot for ventilator‐associated pneumonia.

**FIGURE 7:**
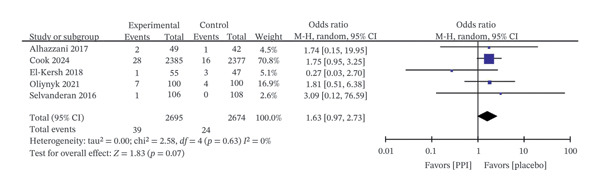
Forest plot for *Clostridioides difficile* infection.

Five trials [18, 23, 26, 28, 29] including 5400 patients reported VAP outcomes, with no significant difference observed between the PPI and control groups (OR: 0.99; 95% CI: 0.87−1.12; I^2^ = 0%; *p* = 0.86) (Figure 6). Likewise, pooled data from five trials [18, 23, 24, 28, 29] with 5369 patients indicated that PPIs did not significantly affect the incidence of *Clostridioides difficile* infection (OR: 1.63; 95% CI: 0.97−2.73; I^2^ = 0%; *p* = 0.07) (Figure 7).

### 4.4. Duration of ICU Stay and Invasive Ventilation

**FIGURE 8:**
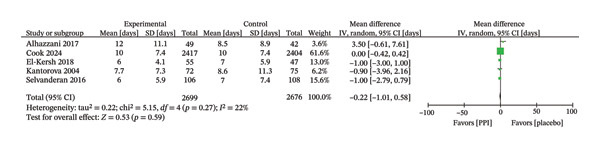
Forest plot for duration of ICU stay.

**FIGURE 9:**
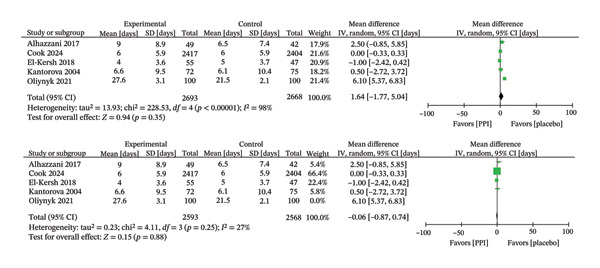
Forest plot for duration of invasive mechanical ventilation.

The duration of ICU stay was analyzed in five trials [18, 23–25, 29], which demonstrated comparable outcomes between the PPI and control groups (MD: −0.22; 95% CI: −1.01−0.58; I^2^ = 22%; *p* = 0.59) (Figure 8). Five trials [18, 23–25, 28] evaluated the duration of invasive ventilation, and the results also showed no statistical difference (MD: 1.64; 95% CI: −1.77−5.04; *p* = 0.35), but substantial heterogeneity was detected among these studies (I^2^ = 98%) (Figure 9). We identified studies that contributed to heterogeneity through sensitivity analysis. After excluding the Oliynyk 2021 study [28], the I^2^ value decreased from 98% to 27%, and the conclusion remained unchanged (MD: −0.06; 95% CI: −0.87−0.74; I^2^ = 27%; *p* = 0.88) (Figure 9).

## 5. Discussion

This systematic review and meta‐analysis confirmed that PPIs significantly reduced the risk of UGIB and clinically important UGIB among patients undergoing invasive ventilation, with no significant effect on mortality. Importantly, there was no evidence that PPIs increased the risk of VAP or *Clostridioides difficile* infection compared to placebo. The durations of ICU stay and invasive ventilation were also comparable between the two groups.

We found that PPIs lowered the risk of UGIB and clinically important UGIB versus placebo or no prophylaxis. The efficacy of PPIs in preventing UGIB in critically ill adults has been confirmed by multiple studies. For example, the REVISE trial, which enrolled 4821 patients, showed that 1.0% of patients in the pantoprazole group experienced clinically important UGIB, compared to 3.5% in the placebo group during ICU stay [18]. A cohort study in Korea found that PPIs can also reduce the risk of UGIB in patients with myocardial infarction (hazard ratio: 0.57; 95% CI: 0.47−0.70; *p* < 0.001) [30]. Furthermore, studies have assessed different classes of drugs for stress ulcer prophylaxis. PPIs demonstrated greater efficacy in reducing the incidence of UGIB compared to H_2_RAs [31–34]. These findings underscore the advantages of PPIs in the prevention of UGIB. The latest guidelines from the United States also recommend that clinicians administer stress ulcer prophylaxis in critically ill adults with risk factors to prevent clinically important UGIB [15].

The lack of effect on mortality indicates that while PPIs reduce UGIB incidence, this does not necessarily translate into improved survival. Similarly, the PEPTIC trial observed no significant difference in mortality between patients receiving PPIs and those receiving H_2_RAs for stress ulcer prophylaxis [31]. Mortality in critically ill patients is multifactorial, and prevention of UGIB may not substantially impact overall mortality rates. Factors such as underlying disease severity, organ dysfunction, and comorbidities often play more significant roles in patient outcomes [35, 36].

Regarding infectious complications, previous studies have raised concerns that acid suppression might promote bacterial overgrowth in the upper gastrointestinal tract, leading to microaspiration and increasing the risk of VAP [37, 38]. In addition, PPIs have been implicated in an increased incidence of *Clostridioides difficile* infection due to alterations in gut microbiota [39, 40]. In a propensity‐matched cohort study of adults receiving invasive ventilation for 24 h or more, PPIs resulted in higher rates of pneumonia (38.6% vs. 27%, *p* < 0.001) and *Clostridioides difficile* infection (3.8% vs. 2.2%, *p* < 0.001) compared to H_2_RAs [19]. However, subsequent high‐quality RCTs largely dispelled this concern. In the SUP‐ICU trial, the incidence of the composite infectious endpoint (pneumonia or *Clostridioides difficile* infection) did not differ significantly between the pantoprazole and placebo groups (16.8% vs. 16.9%; RR: 0.99; 95% CI: 0.84−1.16) [16]. The REVISE trial provided further confirmatory evidence supporting these results [18]. Consistent with randomized evidence, our analyses indicated that the use of PPIs did not significantly increase the risk of VAP or *Clostridioides difficile* infection, nor did it increase ICU length of stay or ventilator use.

An important strength of our meta‐analysis is the incorporation of the recently published REVISE trial [18], which substantially expanded and updated the available evidence on this topic. Nevertheless, certain limitations should be acknowledged when interpreting our results. First, the types of PPIs, dosages, and routes of administration varied across the included studies, possibly introducing heterogeneity in treatment effects. Second, the diagnostic criteria for UGIB and clinically important UGIB were defined differently by study authors, potentially compromising the comparability of results and introducing classification bias. Third, methodological limitations in some studies, particularly the absence of blinding procedures, raise concerns about potential performance and detection biases. Fourth, publication bias was not assessed in this meta‐analysis because only nine studies were included. Assessment of publication bias is generally unreliable when fewer than 10 studies are available, as funnel plots and related statistical tests (e.g., Egger’s test) lack sufficient power to detect bias [41]. Fifth, the incidence of VAP and *Clostridioides difficile* infection was relatively low in the included studies, making estimates imprecise. Furthermore, other effects of PPIs, such as infection with multidrug‐resistant organisms [42] or hypomagnesemia [43], were not reported in these randomized trials.

## 6. Conclusions

In conclusion, prophylactic use of PPIs in mechanically ventilated patients reduces the risk of UGIB and clinically important UGIB without significantly affecting mortality or increasing the risk of VAP and *Clostridioides difficile* infection. Future research should focus on optimal risk stratification, dosing regimens, and treatment duration.

## Author Contributions

Jinlu Hu and Haiyan Ye are co‐first authors. They conducted the study design, literature search, data collection, analysis, and manuscript drafting. Xuemei Zheng assisted in literature retrieval. Bo Li critically evaluated the intellectual content of the manuscript.

## Funding

No funding was received for this study.

## Conflicts of Interest

The authors declare no conflicts of interest.

## Data Availability

All data and materials are available from the corresponding author upon reasonable request.

## References

[1] Cook D. and Guyatt G. , Prophylaxis Against Upper Gastrointestinal Bleeding in Hospitalized Patients, New England Journal of Medicine. (2018) 378, no. 26, 2506–2516, 10.1056/nejmra1605507, 2-s2.0-85049315590. 29949497

[2] Plummer M. P. , Blaser A. R. , and Deane A. M. , Stress Ulceration: Prevalence, Pathology and Association With Adverse Outcomes, Critical Care. (2014) 18, no. 2, 10.1186/cc13780, 2-s2.0-84897395742. PMC4056012 25029573

[3] Cook D. J. , Fuller H. D. , Guyatt G. H. et al., Risk Factors for Gastrointestinal Bleeding in Critically Ill Patients. Canadian Critical Care Trials Group, New England Journal of Medicine. (1994) 330, no. 6, 377–381, 10.1056/nejm199402103300601, 2-s2.0-0028097917. 8284001

[4] Faisy C. , Guerot E. , Diehl J. L. , Iftimovici E. , and Fagon J. Y. , Clinically Significant Gastrointestinal Bleeding in Critically Ill Patients With and Without Stress-Ulcer Prophylaxis, Intensive Care Medicine. (2003) 29, no. 8, 1306–1313, 10.1007/s00134-003-1863-3, 2-s2.0-0042379879. 12830375

[5] Krag M. , Perner A. , Wetterslev J. et al., Prevalence and Outcome of Gastrointestinal Bleeding and Use of Acid Suppressants in Acutely Ill Adult Intensive Care Patients, Intensive Care Medicine. (2015) 41, no. 5, 833–845, 10.1007/s00134-015-3725-1, 2-s2.0-84928801013. 25860444

[6] Cook D. J. , Griffith L. E. , Walter S. D. et al., The Attributable Mortality and Length of Intensive Care Unit Stay of Clinically Important Gastrointestinal Bleeding in Critically Ill Patients, Critical Care. (2001) 5, no. 6, 368–375, 10.1186/cc1071, 2-s2.0-0035543034. 11737927 PMC83859

[7] Krag M. , Perner A. , Wetterslev J. et al., Stress Ulcer Prophylaxis in the Intensive Care Unit: An International Survey of 97 Units in 11 Countries, Acta Anaesthesiologica Scandinavica. (2015) 59, no. 5, 576–585, 10.1111/aas.12508, 2-s2.0-84927740533. 25880349

[8] Shears M. , Alhazzani W. , Marshall J. C. et al., Stress Ulcer Prophylaxis in Critical Illness: A Canadian Survey, Canadian Journal of Anaesthesia. (2016) 63, no. 6, 718–724, 10.1007/s12630-016-0612-3, 2-s2.0-84959126830. 26911559

[9] Barletta J. F. , Kanji S. , MacLaren R. , Lat I. , and Erstad B. L. , Pharmacoepidemiology of Stress Ulcer Prophylaxis in the United States and Canada, Journal of Critical Care. (2014) 29, no. 6, 955–960, 10.1016/j.jcrc.2014.06.025, 2-s2.0-84908145665. 25081626

[10] Dutta A. K. , Jain A. , Jearth V. et al., Guidelines on Optimizing the Use of Proton Pump Inhibitors: PPI Stewardship, Indian Journal of Gastroenterology. (2023) 42, no. 5, 601–628, 10.1007/s12664-023-01428-7. 37698821

[11] Lanas A. , Artal A. , Blás J. M. , Arroyo M. T. , Lopez-Zaborras J. , and Sáinz R. , Effect of Parenteral Omeprazole and Ranitidine on Gastric pH and the Outcome of Bleeding Peptic Ulcer, Journal of Clinical Gastroenterology. (1995) 21, no. 2, 103–106, 10.1097/00004836-199509000-00008, 2-s2.0-0029092979. 8583073

[12] Somberg L. , Morris J. , Fantus R. et al., Intermittent Intravenous Pantoprazole and Continuous Cimetidine Infusion: Effect on Gastric pH Control in Critically Ill Patients at Risk of Developing Stress-Related Mucosal Disease, Journal of Trauma. (2008) 64, no. 5, 1202–1210, 10.1097/ta.0b013e31815e40b5, 2-s2.0-43449108303. 18469642

[13] Ye Z. , Reintam Blaser A. , Lytvyn L. et al., Gastrointestinal Bleeding Prophylaxis for Critically Ill Patients: A Clinical Practice Guideline, BMJ. (2020) 368, 10.1136/bmj.l6722. 31907223

[14] Evans L. , Rhodes A. , Alhazzani W. et al., Surviving Sepsis Campaign: International Guidelines for Management of Sepsis and Septic Shock 2021, Critical Care Medicine. (2021) 49, no. 11, e1063–e1143, 10.1097/ccm.0000000000005337. 34605781

[15] MacLaren R. , Dionne J. C. , Granholm A. et al., Society of Critical Care Medicine and American Society of Health-System Pharmacists Guideline for the Prevention of Stress-Related Gastrointestinal Bleeding in Critically Ill Adults, Critical Care Medicine. (2024) 52, no. 8, e421–e430, 10.1097/ccm.0000000000006330. 39007578

[16] Krag M. , Marker S. , Perner A. et al., Pantoprazole in Patients at Risk for Gastrointestinal Bleeding in the ICU, New England Journal of Medicine. (2018) 379, no. 23, 2199–2208, 10.1056/nejmoa1714919, 2-s2.0-85056307726. 30354950

[17] Granholm A. , Marker S. , Krag M. et al., Heterogeneity of Treatment Effect of Prophylactic Pantoprazole in Adult ICU Patients: A Post Hoc Analysis of the SUP-ICU Trial, Intensive Care Medicine. (2020) 46, no. 4, 717–726, 10.1007/s00134-019-05903-8. 31938829

[18] Cook D. , Deane A. , Lauzier F. et al., Stress Ulcer Prophylaxis During Invasive Mechanical Ventilation, New England Journal of Medicine. (2024) 391, no. 1, 9–20, 10.1056/nejmoa2404245. 38875111

[19] MacLaren R. , Reynolds P. M. , and Allen R. R. , Histamine-2 Receptor Antagonists vs Proton Pump Inhibitors on Gastrointestinal Tract Hemorrhage and Infectious Complications in the Intensive Care Unit, JAMA Internal Medicine. (2014) 174, no. 4, 564–574, 10.1001/jamainternmed.2013.14673, 2-s2.0-84898413831. 24535015

[20] Buendgens L. , Bruensing J. , Matthes M. et al., Administration of Proton Pump Inhibitors in Critically Ill Medical Patients is Associated With Increased Risk of Developing Clostridium Difficile-Associated Diarrhea, Journal of Critical Care. (2014) 29, no. 4, 696.e611–695, 10.1016/j.jcrc.2014.03.002, 2-s2.0-84902272517. 24674763

[21] Page M. J. , McKenzie J. E. , Bossuyt P. M. et al., The PRISMA 2020 Statement: An Updated Guideline for Reporting Systematic Reviews, BMJ. (2021) 372, 10.1136/bmj.n71. PMC8005924 33782057

[22] Higgins J. P. , Altman D. G. , Gøtzsche P. C. et al., The Cochrane Collaboration’s Tool for Assessing Risk of Bias in Randomised Trials, BMJ. (2011) 343, no. oct18 2, 10.1136/bmj.d5928, 2-s2.0-84859001212. PMC3196245 22008217

[23] Alhazzani W. , Guyatt G. , Alshahrani M. et al., Withholding Pantoprazole for Stress Ulcer Prophylaxis in Critically Ill Patients: A Pilot Randomized Clinical Trial and Meta-Analysis, Critical Care Medicine. (2017) 45, no. 7, 1121–1129, 10.1097/ccm.0000000000002461, 2-s2.0-85020492571. 28459708

[24] El-Kersh K. , Jalil B. , McClave S. A. et al., Enteral Nutrition as Stress Ulcer Prophylaxis in Critically Ill Patients: A Randomized Controlled Exploratory Study, Journal of Critical Care. (2018) 43, 108–113, 10.1016/j.jcrc.2017.08.036, 2-s2.0-85035806098. 28865339

[25] Kantorova I. , Svoboda P. , Scheer P. et al., Stress Ulcer Prophylaxis in Critically Ill Patients: A Randomized Controlled Trial, Hepato-Gastroenterology. (2004) 51, no. 57, 757–761. 15143910

[26] Lin C. C. , Hsu Y. L. , Chung C. S. , and Lee T. H. , Stress Ulcer Prophylaxis in Patients Being Weaned From the Ventilator in a Respiratory Care Center: A Randomized Control Trial, Journal of the Formosan Medical Association. (2016) 115, no. 1, 19–24, 10.1016/j.jfma.2014.10.006, 2-s2.0-84958740463. 25676674

[27] Liu B. L. , Li B. , Zhang X. et al., A Randomized Controlled Study Comparing Omeprazole and Cimetidine for the Prophylaxis of Stress-Related Upper Gastrointestinal Bleeding in Patients With Intracerebral Hemorrhage, Journal of Neurosurgery. (2013) 118, no. 1, 115–120, 10.3171/2012.9.jns12170, 2-s2.0-84872172491. 23061387

[28] Oliynyk O. , The Effect of Omeprazole on Treatment Outcomes in Patients With Severe Traumatic Brain Injury and Sepsis, Health Problems of Civilization. (2021) 15, no. 2, 137–141, 10.5114/hpc.2020.98946.

[29] Selvanderan S. P. , Summers M. J. , Finnis M. E. et al., Pantoprazole or Placebo for Stress Ulcer Prophylaxis (POP-UP): Randomized Double-Blind Exploratory Study, Critical Care Medicine. (2016) 44, no. 10, 1842–1850, 10.1097/ccm.0000000000001819, 2-s2.0-84988434139. 27635481

[30] Baik M. , Jeon J. , Kim J. , and Yoo J. , Proton Pump Inhibitor for Gastrointestinal Bleeding in Patients With Myocardial Infarction on Dual-Antiplatelet Therapy: A Nationwide Cohort Study, Journal of Epidemiology and Global Health. (2024) 14, no. 3, 1142–1151, 10.1007/s44197-024-00267-9. 38913256 PMC11442791

[31] Young P. J. , Bagshaw S. M. , Forbes A. B. et al., Effect of Stress Ulcer Prophylaxis With Proton Pump Inhibitors vs Histamine-2 Receptor Blockers on In-Hospital Mortality Among ICU Patients Receiving Invasive Mechanical Ventilation: The PEPTIC Randomized Clinical Trial, JAMA. (2020) 323, no. 7, 616–626, 10.1001/jama.2019.22190. 31950977 PMC7029750

[32] Alhazzani W. , Alshamsi F. , Belley-Cote E. et al., Efficacy and Safety of Stress Ulcer Prophylaxis in Critically Ill Patients: A Network Meta-Analysis of Randomized Trials, Intensive Care Medicine. (2018) 44, no. 1, 1–11, 10.1007/s00134-017-5005-8, 2-s2.0-85036570038. PMC5770505 29199388

[33] Wang Y. , Ge L. , Ye Z. et al., Efficacy and Safety of Gastrointestinal Bleeding Prophylaxis in Critically Ill Patients: An Updated Systematic Review and Network Meta-Analysis of Randomized Trials, Intensive Care Medicine. (2020) 46, no. 11, 1987–2000, 10.1007/s00134-020-06209-w. 32833040

[34] Alshamsi F. , Belley-Cote E. , Cook D. et al., Efficacy and Safety of Proton Pump Inhibitors for Stress Ulcer Prophylaxis in Critically Ill Patients: A Systematic Review and Meta-Analysis of Randomized Trials, Critical Care. (2016) 20, no. 1, 10.1186/s13054-016-1305-6, 2-s2.0-85008656029. PMC4855320 27142116

[35] Melaku E. E. , Urgie B. M. , Dessie F. , Seid A. , Abebe Z. , and Tefera A. S. , Determinants of Mortality of Patients Admitted to the Intensive Care Unit at Debre Berhan Comprehensive Specialized Hospital: A Retrospective Cohort Study, Patient Related Outcome Measures. (2024) 15, 61–70, 10.2147/prom.s450502. 38410832 PMC10895994

[36] Lorencio Cárdenas C. , Yébenes J. C. , Vela E. et al., Trends in Mortality in Septic Patients According to the Different Organ Failure During 15 Years, Critical Care. (2022) 26, no. 1, 10.1186/s13054-022-04176-w. PMC9528124 36192781

[37] Corsonello A. , Lattanzio F. , Bustacchini S. et al., Adverse Events of Proton Pump Inhibitors: Potential Mechanisms, Current Drug Metabolism. (2018) 19, no. 2, 142–154, 10.2174/1389200219666171207125351, 2-s2.0-85046636479. 29219052

[38] Maret-Ouda J. , Panula J. , Santoni G. , Xie S. , and Lagergren J. , Proton Pump Inhibitor Use and Risk of Pneumonia: A Self-Controlled Case Series Study, Journal of Gastroenterology. (2023) 58, no. 8, 734–740, 10.1007/s00535-023-02007-5. 37314495 PMC10366235

[39] Lin C. Y. , Cheng H. T. , Kuo C. J. et al., Proton Pump Inhibitor-Induced Gut Dysbiosis Increases Mortality Rates for Patients With Clostridioides Difficile Infection, Microbiology Spectrum. (2022) 10, no. 4, 10.1128/spectrum.00486-22. PMC9430933 35863023

[40] D′Silva K. M. , Mehta R. , Mitchell M. et al., Proton Pump Inhibitor Use and Risk for Recurrent Clostridioides Difficile Infection: A Systematic Review and Meta-Analysis, Clinical Microbiology and Infections. (2021) . 10.1016/j.cmi.2021.01.008 33465501

[41] Afonso J. , Ramirez-Campillo R. , Clemente F. M. , Büttner F. C. , and Andrade R. , The Perils of Misinterpreting and Misusing “Publication Bias” in Meta-Analyses: An Education Review on Funnel Plot-Based Methods, Sports Medicine. (2024) 54, no. 2, 257–269, 10.1007/s40279-023-01927-9. 37684502 PMC10933152

[42] Willems R. P. J. , Schut M. C. , Kaiser A. M. et al., Association of Proton Pump Inhibitor Use With Risk of Acquiring Drug-Resistant Enterobacterales, JAMA Network Open. (2023) 6, no. 2, 10.1001/jamanetworkopen.2023.0470. PMC9951039 36821114

[43] Begley J. , Smith T. , Barnett K. et al., Proton Pump Inhibitor Associated Hypomagnasaemia–A Cause for Concern?, British Journal of Clinical Pharmacology. (2016) 81, no. 4, 753–758, 10.1111/bcp.12846, 2-s2.0-84956603370. 26613375 PMC4799921

